# MHC2MIL: a novel multiple instance learning based method for MHC-II peptide binding prediction by considering peptide flanking region and residue positions

**DOI:** 10.1186/1471-2164-15-S9-S9

**Published:** 2014-12-08

**Authors:** Yichang Xu, Cheng Luo, Mingjie Qian, Xiaodi Huang, Shanfeng Zhu

**Affiliations:** 1 School of Computer Science, Fudan University, Shanghai 200433, China; 2 Shanghai Key Lab of Intelligent Information Processing, Fudan University, Shanghai 200433, China; 3 Department of Computer Science, University of Illinois at Urbana-Champaign,Urbana, IL 611801, USA; 4 School of Computing and Mathematics, Charles Sturt University, Albury, NSW 2640, Australia

## Abstract

**Background:**

Computational prediction of major histocompatibility complex class II (MHC-II) binding peptides can assist researchers in understanding the mechanism of immune systems and developing peptide based vaccines. Although many computational methods have been proposed, the performance of these methods are far from satisfactory. The difficulty of MHC-II peptide binding prediction comes mainly from the large length variation of binding peptides.

**Methods:**

We develop a novel multiple instance learning based method called MHC2MIL, in order to predict MHC-II binding peptides. We deem each peptide in MHC2MIL as a bag, and some substrings of the peptide as the instances in the bag. Unlike previous multiple instance learning based methods that consider only instances of fixed length 9 (9 amino acids), MHC2MIL is able to deal with instances of both lengths of 9 and 11 (11 amino acids), simultaneously. As such, MHC2MIL incorporates important information in the peptide flanking region. For measuring the distances between different instances, furthermore, MHC2MIL explicitly highlights the amino acids in some important positions.

**Results:**

Experimental results on a benchmark dataset have shown that, the performance of MHC2MIL is significantly improved by considering the instances of both 9 and 11 amino acids, as well as by emphasizing amino acids at key positions in the instance. The results are consistent with those reported in the literature on MHC-II peptide binding. In addition to five important positions (1, 4, 6, 7 and 9) for HLA(human leukocyte antigen, the name of MHC in Humans) DR peptide binding, we also find that position 2 may play some roles in the binding process. By using 5-fold cross validation on the benchmark dataset, MHC2MIL outperforms two state-of-the-art methods of MHC2SK and NN-align with being statistically significant, on 12 HLA DP and DQ molecules. In addition, it achieves comparable performance with MHC2SK and NN-align on 14 HLA DR molecules. MHC2MIL is freely available at http://datamining-iip.fudan.edu.cn/service/MHC2MIL/index.html.

## Background

Major Histocompatibility Complex (MHC) molecules play important roles in adaptive immune response. An important function of MHC molecules is to bind peptide fragments derived from pathogens and to display them on the cell surface for being recognized by appropriate T cells [1]. This stimulates subsequent immune response in order to fight against these pathogens. The MHC gene family is mainly divided into two subgroups: class I (MHC-I) and class II (MHC-II). Peptides presented by MHC-I originate from proteins produced within a cell, while those by MHC-II are from the outside of the cell. T helper cells (one type of T cells), which are activated by the peptides presented by MHC-II molecules, control or help the immune response. Therefore, accurate identification of MHC-II binding peptides is crucial in understanding the mechanism of immune systems, as well as in developing peptide-based vaccines for treating some serious diseases, such as hepatitis, EB virus, autoimmunity, and cancer [2]. Compared to biochemical experiments with high costs in both time and finance, computational methods are more economic in that they can be quickly deployed to select a small number of promising peptides for further biochemical experimental verification.

Computational methods for predicting MHC-I binding peptides are reported to produce considerable results of AUC (Area Under ROC Curve) between 0.85 and 0.95 [2]. This is because the prediction of MHC-I binding peptides is relatively easy, where the binding groove of MHC-I molecules is closed at both ends and the binding peptides usually have a length between 8 and 11 amino acids [1]. However, the prediction of MHC-II binding peptides is more challenging. One main reason for this is that the binding groove of MHC-II molecules is open at both ends, which leads that the MHC-II binding peptides typically vary from 11 to 20 amino acids in length [3]. Usually MHC peptide binding core is of 9 amino acids that fit into 9 pockets of MHC binding groove. Although a number of computational methods has been developed to predict MHC-II binding peptides in the last few years [4-18], recent experimental results on various benchmark datasets show that the performance of these methods needs to be improved [2,19]. These computational methods can be divided into two groups: allele-specific and pan-specific methods [2]. In the allele-specific methods, both training and test peptides are for a same MHC molecule. In contrast, pan-specific methods can predict the binding peptides of MHC molecules that have very few or even no training data [2,20,21]. In this work, we focus on allele-specific methods that are the basis of pan-specific methods.

According to underlying techniques used, the allele-specific methods can be roughly divided into four groups: position specific score matrix (PSSM) based methods, artificial neural network (ANN) based methods, kernel based methods, and multiple instance learning based methods. Although TEPITOPE [10], ARB [8], CombLib [9], TEPITOPEpan [17] and SMM-align [4] are all PSSM based methods, they differ significantly in the way of generating the score matrices. TEPITOPE is the first PSSM method in which the score matrix is obtained by examining the binding specificities of target MHC molecule in each pocket using biochemical experiments. The PSSM of ARB is modeled by the difference of average binding affinity among 6 residue groups. The CombLib method derives the matrix from a positional scanning combinatorial libraries consisting of 180 peptides for each MHC molecule. TEPITOPEpan extends TEPITOPE by covering all HLA-DR molecules instead of only 51 HLA-DR molecules. In SMM-align, a Metropolis Monte Carlo procedure is employed to search for an optimal score matrix from the binding affinity data [4]. Unlike these PSSM based methods, NN-align [5] is an ANN based method. It first estimates the peptide binding core, then this part and flanking region are co-encoded and input into ANN in order to learn a prediction model. Finally, an ensemble of ANN is used to improve the robustness of the method. To avoid the difficulty of estimating the binding core, several kernel based methods have been proposed to directly measure the similarities among peptides of different lengths. Local alignment (LA) kernel [7] makes use of the alignment method for similarity calculations, while GS [22] and MHC2SK [18] are two alignment free methods that use a series of substrings to measure peptide similarities. One notable difference between GS and MHC2SK is that MHC2SK emphasizes long substrings while GS considers a substring whose length is as short as 1. Several recent methods resort to a multiple instance learning (MIL) framework to handle the length variation of peptides. In this framework, each pepitde is regarded as a bag, and some substrings of the peptide as the instances in the bag. Two MIL based methods, MHCMIR [16] and MultiMHCII [13], have been proposed to tackle the problem of MHC-II peptide binding prediction. Both of them create bags with 9 amino acids (AA) long instances and use Support Vector Regression (SVR) as the predictor. MultiMHCII uses a normalized set kernel as its kernel function and MHCMIR is an adaption of MILES [23].

Although both MHCMIR and MultiMHCII can deal with the length variation of binding peptides, some important peptide information has been ignored in their modeling process. First, in addition to the binding core region (9 amino acids), the peptide flanking region also contributes the binding process. However, both MHCMIR and MutliMHCII consider only instances of 9 AA, where the effect of the peptide flanking region could not be modeled. Second, it has been reported that the amino acids at some specific positions (e.g. 1, 4, 6, 7, 9) of the peptide binding core are more crucial for the binding process [10]. This kind of important domain knowledge is not well incorporated into MHCMIR and MultiMHCII for improving the prediction performance. To address these concerns, we develop a novel MHC-II multiple instance learning based method, MHC2MIL, that makes use of these important domain knowledge. Unlike MHCMIR and MultiMHCII, both length 9 and 11 instances are considered in our MHC2MIL so as to integrate useful information in both potential binding cores and peptide flanking region. We use a benchmark data covering 26 HLA DR, DP, and DQ molecules to evaluate the performance of MHC2MIL. Experimental results demonstrate the effectiveness of incorporating peptide flanking region, as well as the importance of some crucial pepitde positions. Interestingly, the experimental results show that position 2 may also play some roles in HLA-DR peptide binding process. Furthermore, the performance of MHC2MIL is compared with two state-of-the-art methods of MHC2SK and NN-align. MHC2MIL outperforms both MHC2SK and NN-align with being statistically significant on 12 HLA DP and DQ molecules. In addition, it achieves the comparable performance to MHC2SK and NN-align on 14 HLA DR molecules.

## Materials and methods

### Data

We use a benchmark dataset (Wang dataset) described in [9] to compare the performance of different computational methods. This dataset is of high quality, which has been widely used in the evaluation of different computational methods. As shown in Table 1, the dataset consists of 24382 peptides with binding affinities covering 26 different HLA DR, DP and DQ molecules. Each HLA molecule has the sufficient number of peptides with binding affinity, ranging from 474 (HLA-DRB1_0404) to 3504 (HLA-DRB1_0101). The dataset has also been divided into five folds with the similar size.

**Table 1:** Statistics for Wang SR. Peptides with IC50 less than 1000 being deemed as binders

Allele	Count	Binder	Non-Binder
HLA-DPA1_01-DPB1_0401	540	150	390
HLA-DPA1_0103-DPB1_0201	603	203	400
HLA-DPA1_0201-DPB1_0101	604	245	359
HLA-DPA1_0201-DPB1_0501	586	163	423
HLA-DPA1_0301-DPB1_0402	602	210	392
HLA-DPB1_0301-DPB1_0401	549	161	388
HLA-DQA1_0101-DQB1_0501	584	141	443
HLA-DQA1_0102-DQB1_0602	593	287	306
HLA-DQA1_0301-DQB1_0302	596	178	418
HLA-DQA1_0401-DQB1_0402	585	146	439
HLA-DQA1_0501-DQB1_0201	589	159	430
HLA-DQA1_0501-DQB1_0301	602	355	247
HLA-DRB1_0101	3504	2347	1157
HLA-DRB1_0301	1136	440	696
HLA-DRB1_0401	1221	695	526
HLA-DRB1_0404	474	317	157
HLA-DRB1_0405	1049	585	464
HLA-DRB1_0701	1175	726	449
HLA-DRB1_0802	1017	426	591
HLA-DRB1_0901	1042	569	473
HLA-DRB1_1101	1204	682	522
HLA-DRB1_1302	1070	471	599
HLA-DRB1_1501	1171	645	526
HLA-DRB3_0101	987	317	670
HLA-DRB4_0101	1011	489	522
HLA-DRB5_0101	1198	710	488

All	24382	11817	12475

### Method

#### Multiple instance learning

Multiple Instance Learning (MIL) is first introduced in 1997 [24]. Differing from classic learning methods, MIL uses "bags" instead of "instances". A bag consists of one or more instances. Note that a bag is positive if at least one of its instances is positive, and negative, otherwise. We denote *B *as a set of bags, *B_i _*as the *ith *bag in *B*, and *B_ij _*as the *jth *instances in *B_i_*. Function *Label *maps a bag or a instance into {-1, +1}. Then the label of a bag is as follows:

(1) $$Label\left(B_{i}\right)=-1\;if\;f.\;\forall B_{ij},\;\;Label\left(B_{ij}\right)=-1$$

(2) $$Label\left(B_{i}\right)=1\;\exists B_{ij},\;Label\left(Bi_{j}\right)=1$$

where *i f f *. stands for "if and only if". If a bag is positive, we would not know which instance (instances) is (are) positive.

The MIL method of Axis-Parallel Rectangles (APR) [24] tries to find a rectangle that contains at least one instance from each positive bag while no instances from negative bags are included. Unfortunately, this kind of rectangle may not always exist. The famous MIL algorithm of Diverse Density (DD) [25] attempts to find a concept *t *with the highest "diverse density" instead of a rectangle. Such *t *is close to positive bags and far from negative bags. If we use $B_{i}^{+}$ to stand for the *ith *positive bag, and $B_{i}^{-}$ for the *ith *negative bag, then DD maximizes the equation:

(3) $$DD\left(t\right)=\underset{i}{\prod }Pr\left(t\text{|}B_{i}^{+}\right)\;\;\underset{i}{\prod }Pr\left(t\text{|}B_{i}^{-}\right)$$

Multiple-instance learning via embedded instance selection (MILES) extends DD by considering that the DD framework appears to be rather restrictive because it always seeks for one and only one feature in feature selection. DD can be improved by searching for multiple features [23]. In MILES, all instances from bags are shuffled to a meta-space *C *for mapping each bag into a feature:

(4) $$C=\left\{x^{k}:k=1,2,...,n\right\}$$

where *x^k ^*is a re-indexed instance from all of the bags and *n *is the total number of instances. Based on the meta-space, the probability of generating *x^k ^*from *B_i _*is

(5) $$Pr(x^{k}|Bi)=s(x^{k},B_{i})=\underset{j}{\max}\exp\left(-\frac{||B_{ij}-x^{k}|{|}^{2}}{\sigma^{2}}\right)$$

where *s*(*x^k ^, B_i_*) can be interpreted as a measure of the similarity between *x^k ^*and *B_i_*. The similarity is determined by the closest instance to *x^k ^*in *B_i_*. Then bag *B_i _*is mapped to a vector of features:

(6) $$m\left(B_{i}\right)={\left[s\left(x^{1},B_{i}\right),s\left(x^{2},B_{i}\right),...,s\left(x^{n},B_{i}\right)\right]}^{T}$$

MILES maps each bag into the feature space and applies SVM as the classifier. The essence of MILES is that if examples cannot be separated in the low dimension, they are mapped into high dimension space. If *x^k ^*has a high similarity to some positive bags and a low similarity to some negative bags, it would provide useful information in the classification.

### MHCMIR

Based on the general assumption that the peptide binding core is of 9 AA, MIL can be applied to the prediction of MHC-II binding peptides by creating a bag with 9-long instances. We denote *p *as an original peptide, *p*(*i*) as the *ith *amino acid in the *p*, and *len*(*p*) as the total number of amino acids. A bag *B_i _*corresponding to *p *is generated as follows:

(7) $$B_{i}=\{s\text{|}s=p\left(i\right)p\left(i+1\right)...p\left(i+8\right),1\leq i\leq \;len\left(p\right)-8\}$$

MHCMIR adapts MILES to this problem after creating bags, by (1) replacing SVM with SVR; and (2) using matrix BLOSUM62 to compute the similarity between a bag and an instance [23]. Equation 5 is rewritten as:

(8) $$s\left(x^{k},B_{i}\right)=\underset{j}{\text{min}}dis\left(x^{k},B_{ij}\right)$$

where *dis *function is computed as follows:

(9) $$d\left(x,x\prime \right)=\overset{9}{\underset{i=1}{\sum }}BLOS\;UM62\left(x\left(i\right),x\prime \left(i\right)\right)$$

(10) $$dis\left(x,x\prime \right)=\left\{\begin{array}{cc} \frac{1}{d\left(x,x\prime \right)}, & d\left(x,x\prime \right)>0\\ 1, & d\left(x,x\prime \right)\leq 0 \end{array}\right)$$

*x*(*i*) in Equation 9 is the *ith *amino acid of instance *x*. The distance between two 9-long instances is computed by two steps: calculating the sum of the corresponding positions in BLOSUM62, and scaling the value obtained in step one to (0, 1] according to its sign.

### MHC2MIL

The critical drawback of MHCMIR is that important domain knowledge, such as the effect of peptide flanking region and the importance of key positions in binding core, are ignored in the modelling. To address this shortcoming, we extends MHCMIR to MHC2MIL by taking into account two facts:

(1) A bag can have instances with different lengths; and

(2) Key positions of an instance should be emphasized.

Specifically, we create bags to include both 9-long and 11-long instances. The assumption is that the information from the left and right amino acids of the 9-long instance is useful, and meta-space is expanded by adding 11-long instances, both of which are benefit to the prediction. In addition, according to related literature on MHC-II peptide binding, positions 1, 4, 6, 7 and 9 are the most influential for binding process. Therefore we use the amino acids at these positions instead of all positions to measure the distance between two instances.

Thus Equation 9 is modified as:

(11) $$d\left(x,x\prime \right)=\left\{\begin{array}{cc} \sum_{i\in KP_{9}}BLOS\;UM62\left(x\left(i\right),x\prime \left(i\right)\right), & len\left(x\right)=len\left(x\prime \right)=9;\\ \sum_{i\in KP11}BLOS\;UM62\left(x\left(i\right),x\prime \left(i\right)\right), & len\left(x\right)=len\left(x\prime \right)=11;\\ \infty , & otherwise. \end{array}\right)$$

where *KP*_9 _= {1, 4, 6, 7, 9} is the key positions for 9-long instances and *KP*_11 _= {1, 2, 5, 7, 8, 10, 11} for 11-long instances. According to our assumption, an 11-long instance is its middle 9-long instance with the left and right amino acids, so the first, last positions and 1,4,6,7,9 positions of the middle 9-long instance are taken into consideration. For the sake of simplicity, we assign ∞ for the distance between instances with different lengths.

## Results and discussion

### Experimental procedure and evaluation metric

According to [9], Wang's dataset was divided into 5 partitions, and the peptide with the binding affinity of less than 1000 nM was deemed as a binder. We thus validated our model by 5-fold cross validation. For training MHC2MIL, similar to [5], the binding value was transformed by 1 - *log*(*IC*50)/*log*(50, 000), where IC50 is binding affinity measured in nM. libsvm [26] was used for our implementation of SVR in MHC2MIL and MHCMIR. The parameters *c *and *g *of SVR were set as the default values where *c *= 1 and *g *= 1/#*f eatures *(#*f eatures *is the total number of the input features). We implemented MHC2SK according to the related paper [18]. AUC was used as the evaluation metric to compare different models. In addition, for comparing two predictors, we used one-tailed per-allele binomial test to measure their performance differences, where *p *- *value *< 0.05 is considered to being statistically significant. In the following, we denote MHC2MIL as MHC2MIL(fl) that considers instances of flexible length (both 9-long and 11-long instances), and MHC2MIL as MHC2MIL(fl+kp) that considers both instances of flexible length and key positions.

### Effect of adding 11-long instances

First, we examined the effect of incorporating both 9-long and 11-long instances into bags. Comparisons were done among MHCMIR(9), MHCMIR(11), and MHC2MIL(fl),where MHCMIR(*n*) stands for filling bags with *n *AA long instances. The results are given in Table 2. In all 26 alleles, the best performance is achieved by MHCMIR(9) on 2 alleles, MHCMIR(11) on 7 alleles, and MHC2MIL(fl) on 20 alleles. MHC2MIL(fl) achieved the highest average AUC of 0.777, outperforming both MHCMIR(9) (AUC of 0.770) and MHCMIR(11) (AUC of 0.771) with being statistically significant (binomial test, *p *- *value *< 0.05). From this we can see that addition of 11-long instances into bags provides useful information to improve the prediction performance. This is consistent with previous studies in that not only the binding core but also peptide flanking region contribute to the binding process.

**Table 2:** Performance of MHC2MIL(fl) compared to MHCMIR(9) and MHCMIR(11). For each allele, the largest value is displayed in boldface.

allele	MHCMIR(9)	MHCMIR(11)	MHC2MIL(fl)
HLA-DPA1_01-DPB1_0401	0.772	**0.780**	0.778
HLA-DPA1_0103-DPB1_0201	0.782	**0.792**	0.790
HLA-DPA1_0201-DPB1_0101	0.810	0.811	**0.817**
HLA-DPA1_0201-DPB1_0501	0.784	0.790	**0.791**
HLA-DPA1_0301-DPB1_0402	0.820	**0.832**	**0.832**
HLA-DPB1_0301-DPB1_0401	0.785	0.782	**0.791**
HLA-DQA1_0101-DQB1_0501	0.763	0.761	**0.768**
HLA-DQA1_0102-DQB1_0602	0.751	0.760	**0.763**
HLA-DQA1_0301-DQB1_0302	0.666	0.665	**0.672**
HLA-DQA1_0401-DQB1_0402	0.732	0.721	**0.737**
HLA-DQA1_0501-DQB1_0201	0.772	0.758	**0.779**
HLA-DQA1_0501-DQB1_0301	0.804	0.800	**0.809**
HLA-DRB1_0101	0.770	**0.789**	0.786
HLA-DRB1_0301	0.793	0.805	**0.808**
HLA-DRB1_0401	0.738	0.735	**0.741**
HLA-DRB1_0404	**0.793**	0.773	0.791
HLA-DRB1_0405	0.790	0.784	**0.792**
HLA-DRB1_0701	0.809	**0.819**	**0.819**
HLA-DRB1_0802	0.700	**0.717**	0.712
HLA-DRB1_0901	0.730	0.723	**0.733**
HLA-DRB1_1101	0.831	0.825	**0.834**
HLA-DRB1_1302	0.736	0.741	**0.745**
HLA-DRB1_1501	0.752	**0.767**	0.765
HLA-DRB3_0101	0.749	0.740	**0.756**
HLA-DRB4_0101	**0.787**	0.776	**0.787**
HLA-DRB5_0101	0.796	0.792	**0.803**

average AUC	0.770	0.771	**0.777**

### Effect of first position limitation on HLA DR

Second, we explored the effect of adding first position limitation on generating instances for HLA DR molecules. Previous work suggests that a 9-long peptide in HLA DR could become a binding core only when aliphatic (I, L, M, V) and aromatic (F, W, Y) amino acids appear in its first position [10]. We can reduce the instances in a bag by adding a constraint that the first position of the instance should be in (I, L, M, V, F, W, Y). The comparison results are shown in Table 3, where MHC2MIL(fl+fpl) denotes the method that creates bags by utilizing first position limitation based on MHC2MIL(fl). Compared with MHC2MIL(fl), MHC2MIL(fl+fpl) performed worse in all alleles. The average AUC was reduced greatly from 0.777 to 0.734. This suggests that reducing too many negative instances may lose some important information for discriminating binders from non-binders.

**Table 3:** Performance of MHC2MIL(fl) compared to MHC2MIL(fl_fpl). For each allele, the largest value is displayed in boldface.

allele	MHC2MIL(fl)	MHC2MIL(fl_fpl)
HLA-DRB1_0101	**0.786**	0.745
HLA-DRB1_0301	**0.808**	0.765
HLA-DRB1_0401	**0.741**	0.697
HLA-DRB1_0404	**0.791**	0.771
HLA-DRB1_0405	**0.792**	0.737
HLA-DRB1_0701	**0.819**	0.783
HLA-DRB1_0802	**0.712**	0.688
HLA-DRB1_0901	**0.733**	0.692
HLA-DRB1_1101	**0.834**	0.757
HLA-DRB1_1302	**0.745**	0.708
HLA-DRB1_1501	**0.765**	0.718
HLA-DRB3_0101	**0.756**	0.709
HLA-DRB4_0101	**0.787**	0.760
HLA-DRB5_0101	**0.803**	0.746

average AUC	**0.777**	0.734

### Effect of utilizing key positions

Finally, we examined the effect of emphasizing some key positions of instances. According to Equation 11, MHC2MIL(fl+kp) computes the distance between two instances based on only key positions. Table 4 compares the performance of MHC2MIL(fl+kp) to that of MHC2MIL(fl). MHC2MIL(fl+kp) achieved a higher average AUC of 0.784, while MHC2MIL(fl) achieved a lower average AUC of 0.777. Overall, MHC2MIL(fl+kp) outperformed MHC2MIL(fl) in 19 out of all 26 alleles, with being statistically significant (binomial test, *p *- *value *< 0.05). This indicates that those key positions are more influential than other positions in the binding process. Furthermore, a close analysis of results indicates that, compared with HLA DR molecules, HLA DP and DQ molecules benefit more from utilizing these key positions. Out of all 12 HLA DP and DQ molecules, the prediction performances of 11 were improved by emphasizing key positions. On the other hand, MHC2MIL(fl+kp) outperformed MHC2MIL(fl) in only 8 out of all 14 HLA DR molecules. We hypothesize that some other positions may play some roles in the HLA DR binding process. We added the left 2,3,5,and 8 positions to Equation 11 respectively to see the effect of these positions on HLA DR. The experimental results were reported in Table 5, where MHC2MIL(+n) means that position *n *is added into key positions, such as, in MHC2MIL(+2), *KP*_9 _= {1, 2, 4, 6, 7, 9} and *KP*_11 _= {1, 2, 3, 5, 7, 8, 10, 11}. From the experimental results, we can see that MHC2MIL(+2) is the best prediction method in 11 out of all 14 alleles, which achieved the highest average AUC of 0.780. Specifically, MHC2MIL(+2) outperformed MHC2MIL(fl) in 11 out of 14 alleles (with one tie), and MHC2MIL(fl+kp) in all 14 alleles. Both of them are statistically significant (binomial test, *p *- *value *< 0.05). This indicates that the position 2 on DR may be another important binding position besides well-known key positions of 1, 4, 6, 7 and 9.

**Table 4:** Performance of MHC2MIL(fl) compared to MHC2MIL(fl+kp). For each allele, the largest value is displayed in boldface.

allele	MHC2MIL(fl)	MHC2MIL(fl+kp)
HLA-DPA1_01-DPB1_0401	0.778	**0.791**
HLA-DPA1_0103-DPB1_0201	0.790	**0.808**
HLA-DPA1_0201-DPB1_0101	0.817	**0.834**
HLA-DPA1_0201-DPB1_0501	0.791	**0.805**
HLA-DPA1_0301-DPB1_0402	0.832	**0.846**
HLA-DPB1_0301-DPB1_0401	**0.791**	0.790
HLA-DQA1_0101-DQB1_0501	0.768	**0.781**
HLA-DQA1_0102-DQB1_0602	0.763	**0.769**
HLA-DQA1_0301-DQB1_0302	0.672	**0.706**
HLA-DQA1_0401-DQB1_0402	0.737	**0.774**
HLA-DQA1_0501-DQB1_0201	0.779	**0.810**
HLA-DQA1_0501-DQB1_0301	0.809	**0.817**
HLA-DRB1_0101	0.786	**0.787**
HLA-DRB1_0301	**0.808**	0.803
HLA-DRB1_0401	**0.741**	0.732
HLA-DRB1_0404	**0.791**	0.782
HLA-DRB1_0405	**0.792**	0.785
HLA-DRB1_0701	0.819	**0.823**
HLA-DRB1_0802	0.712	**0.716**
HLA-DRB1_0901	0.733	**0.735**
HLA-DRB1_1101	0.834	**0.838**
HLA-DRB1_1302	**0.745**	0.734
HLA-DRB1_1501	**0.765**	0.763
HLA-DRB3_0101	0.756	**0.757**
HLA-DRB4_0101	0.787	**0.792**
HLA-DRB5_0101	0.803	**0.808**

average AUC	0.777	**0.784**

**Table 5:** Performance of adding 2,3,5,8 positions respectively to MHC2MIL(fl+kp) on DR. For each allele, the largest value is displayed in boldface.

allele	MHC2MIL(fl)	MHC2MIL(fl+kp)	MHC2MIL(+2)	MHC2MIL(+3)	MHC2MIL(+5)	MHC2MIL(+8)
HLA-DRB1_0101	0.786	0.787	**0.792**	0.787	0.785	0.785
HLA-DRB1_0301	0.808	0.803	**0.809**	0.807	0.799	0.806
HLA-DRB1_0401	**0.741**	0.732	0.736	0.738	0.727	0.734
HLA-DRB1_0404	**0.791**	0.782	0.786	0.783	0.773	0.775
HLA-DRB1_0405	**0.792**	0.785	**0.792**	0.786	0.790	0.785
HLA-DRB1_0701	0.819	0.823	**0.825**	0.823	0.823	0.821
HLA-DRB1_0802	0.712	0.716	0.718	0.715	0.720	**0.721**
HLA-DRB1_0901	0.733	0.735	**0.736**	0.734	0.731	0.728
HLA-DRB1_1101	0.834	0.838	**0.843**	0.842	0.838	0.837
HLA-DRB1_1302	0.745	0.734	**0.746**	0.744	0.733	0.735
HLA-DRB1_1501	0.765	0.763	**0.770**	0.766	0.764	0.759
HLA-DRB3_0101	0.756	0.757	**0.762**	0.755	0.750	0.749
HLA-DRB4_0101	0.787	0.792	**0.797**	0.791	0.790	0.788
HLA-DRB5_0101	0.803	0.808	**0.811**	0.806	0.799	0.805

average AUC	0.777	0.775	**0.780**	0.777	0.773	0.773

### Comparison with other allele-specific methods

In this section, we present the performance comparisons of MHC2MIL with other allele-specific methods against Wang's dataset using five fold cross validation. The results on HLA DP and DQ are shown in Table 6, and those on HLA DR in Table 7. For HLA DR, position 2 is also deemed as a key position in the implementation of MHC2MIL. There are total 7 allele specific methods of ARB, SMM-align, CombLib, TEPITOPE, MHC2SK, NN-align, and MHC2MIL. CombLib is only for HLA DP and DQ, while TEPITOPE is only for HLA DR. Since we used the same dataset, partition, and experimental procedure, the experimental results of ARB, SMM-align, CombLib, TEPITOPE and NN-align were directly taken from [9]. As shown in their paper [13], MultiMHCII's performance is as low as TEPITOPE, so we did not compare this method. As given in Table 6, MHC2MIL, MHC2SK and NN-align are three best prediction methods for HLA DP and DQ molecules with the highest average AUC of 0.794, 0.783 and 0.783, respectively. In particular, MHC2MIL outperformed NN-align in 9 out of 11 alleles, and MHC2SK in 9 out of 12 alleles, both of which are being statistically significant (binomial test, *p *- *value *< 0.05). For HLA DR molecules, as shown in Table 7, MHC2MIL, MHC2SK and NN-align are also the three best prediction methods with the highest average AUC of 0.780, 0.780 and 0.776 respectively. Specifically, MHC2MIL outperformed NN-align in 9 out of 14 alleles, and MHC2SK in 5 out of 14 alleles (with one tie). Overall, MHC2MIL is the best prediction method for HLA DP and DQ molecules, and achieved comparable performance with two state-of-the-art methods of MHC2SK and NN-align on HLA DR molecules.

**Table 6:** Comparisons of ARB, SMM-align, CombLib, MHC2SK, NN-algin and MHC2MIL on DP and DQ. For each allele, the largest value is displayed in boldface.

allele	ARB	SMM-align	CombLib	MHC2SK	NN-align	MHC2MIL
HLA-DPA1_01-DPB1_0401	0.746	0.767	0.704	0.778	**0.802**	0.791
HLA-DPA1_0103-DPB1_0201	0.745	0.767	0.724	0.789	0.793	**0.808**
HLA-DPA1_0201-DPB1_0101	0.743	0.786	0.723	0.824	0.818	**0.834**
HLA-DPA1_0201-DPB1_0501	0.709	0.728	0.729	0.794	0.787	**0.805**
HLA-DPA1_0301-DPB1_0402	0.771	0.818	0.756	0.838	0.828	**0.846**
HLA-DPB1_0301-DPB1_0401	-	-	-	**0.792**	-	0.790
HLA-DQA1_0101-DQB1_0501	0.741	0.783	0.728	0.789	**0.805**	0.781
HLA-DQA1_0102-DQB1_0602	0.708	0.734	0.752	**0.770**	0.762	0.769
HLA-DQA1_0301-DQB1_0302	0.637	0.663	0.616	0.676	0.693	**0.706**
HLA-DQA1_0401-DQB1_0402	0.643	0.761	0.637	0.767	0.742	**0.774**
HLA-DQA1_0501-DQB1_0201	0.700	0.736	0.620	0.775	0.777	**0.810**
HLA-DQA1_0501-DQB1_0301	0.756	0.801	0.745	0.807	0.811	**0.817**

average AUC	0.718	0.759	0.703	0.783	0.783	**0.794**

**Table 7:** Comparisons of ARB, SMM-align, PROPRED, MHC2SK, NN-algin and MHC2MIL on DR. For each allele, the largest value is displayed in boldface.

allele	ARB	SMM-align	PROPRED	MHC2SK	NN-align	MHC2MIL(+2)
HLA-DRB1_0101	0.710	0.756	0.692	**0.792**	0.763	**0.792**
HLA-DRB1_0301	0.728	0.808	0.669	0.812	**0.829**	0.809
HLA-DRB1_0401	0.668	0.721	0.711	0.726	0.734	**0.736**
HLA-DRB1_0404	0.681	0.789	0.753	0.791	**0.803**	0.786
HLA-DRB1_0405	0.716	0.767	0.742	0.783	**0.794**	0.792
HLA-DRB1_0701	0.736	0.796	0.750	**0.827**	0.811	0.825
HLA-DRB1_0802	0.649	0.689	0.641	**0.719**	0.698	0.718
HLA-DRB1_0901	0.654	0.696	-	**0.744**	0.713	0.736
HLA-DRB1_1101	0.777	0.829	0.779	0.844	**0.847**	0.843
HLA-DRB1_1302	0.667	**0.754**	0.577	0.729	0.732	0.746
HLA-DRB1_1501	0.696	0.741	0.703	**0.771**	0.756	0.770
HLA-DRB3_0101	0.678	0.780	-	0.759	**0.798**	0.762
HLA-DRB4_0101	0.747	0.762	-	**0.809**	0.789	0.797
HLA-DRB5_0101	0.697	0.776	0.711	0.807	0.795	**0.811**

average AUC	0.700	0.762	0.703	**0.780**	0.776	**0.780**

As shown in Table 8, we present the prediction binding affinities (IC50nM) of 5 peptides to HLA-DQA1_0501- DQB1_0201 in Table 8 by MHC2MIL, MHC2SK and NN-align, respectively. For all these 5 peptides, MHC2MIL achieved the closest binding affinity, compared with MHC2SK and NN-align. For example, for peptide "SVLLVVALFAVFLGS" of binding affinity of 748.1nM, the predicted affinity by MHC2MIL is 745nM, while the predicted values by MH2SK and NN-align are 1072.4nM and 2064.6nM, respectively. All these results demonstrate the effectiveness of MHC2MIL on predicting MHC-II binding peptides.

**Table 8:** Predicting values of some peptides by MHC2SK, NN-algin and MHC2MIL on HLA-DQA1_0501-DQB1_0201. IC50_t is the true IC50(nM) for each peptide. IC50_p is the predicting value.

peptide	IC50_t	IC50_p by MHC2SK	IC50_p by NN-algin	IC50_p by MHC2MIL
EDVGYPIIIDQKYCP	849.6	1245.3	1764.5	868.2
FEAMYLGTCQTLTPM	670.8	620.9	1473.4	667.8
LGHRDALEDDLLNRN	1874.1	2275.0	775.5	1844.2
QLVPKLDEVYNAAYN	1478.8	3770.5	994.7	1417.3
SVLLVVALFAVFLGS	748.1	1072.4	2064.6	745.0

### Discussion

Although multiple instance learning has been already applied in MHCMIR and MultiMHCII, the domain knowledge in MHC-II peptide binding is not incorporated very well. MHCMIR and MultiMHCII consider only 9 length instances and MHCMIL treats 9 amino acids in each instances equally. In contrast, MHC2MIL incorporates both length 9 and 11 instances to measure the effect of flanking regions. In addition, only the amino acids in the key positions of the instance are used to measure the distance between two instances in MHC2MIL. It is not surprising that MHC2MIL outperformed MHCMIR on the benchmark dataset, with being statistically significant. In fact, previous studies show that the performance of MHCMIR is comparable with SMM-align [16], and MultiMHCII with TEPITOPE [13]. In contrast, our experimental results demonstrate that, MHC2MIL is the best performed method in HLA DP and DQ molecules out of all six well-known computational methods, achieving comparable performance on HLA DR molecules with two state-of-the-art computational methods of NN-align and MHC2SK. All these indicate that incorporating domain knowledge into the algorithm design is able to greatly boost the prediction accuracy. On the other hand, some interesting knowledge can be captured from the data. In this work, we find that, for HLA DR molecules, in addition to 5 well-known key positions (1, 4, 6, 7 and 9), position 2 may also play some roles in MHC-II pepitde binding process. In fact, according to the position specific scoring matrix provided by TEPITOPE, both positions 2 and 3 contribute slightly to the HLA DR binding process. More experimental studies should be carried out to elucidate the role of these two positions.

Our experimental results suggest that, for the MHC-II pepitde binding prediction, MHC2MIL, MH2SK and NNalign are the three best predicting methods. Differing from NN-align using multiple neural network ensembles, both MHC2SK and MHC2MIL use a single classifier. Moreover, the underlying principles of these algorithms are quite different. For NN-align, the binding core is estimated and encoded with peptide flanking region as a vector. Neither MHC2MIL nor MHC2SK needs to estimate the binding core. MHC2SK relies on string kernel based methods, where short substrings of varying sizes are used to measure the similarity between two peptides. MHC2MIL resorts to multiple instance learning techniques to map each peptide into a common metaspace. It is obvious that these methods are complement to each other, so that ensemble techniques could be used to further enhance the prediction performance [27,28].

## Conclusion

In this paper, we have presented a novel MIL algorithm called MHC2MIL that predicts MHC-II binding peptides by creating bags of flexible instances and utilizing key positions to calculate the similarity. MHC2MIL achieves competitive results with two state-of-the-art computational approaches of MHC2SK and NN-align. Considering the high diversity of MHC-II molecules, we would extend MHC2MIL to be a pan-specific algorithm in order to cover many more MHC-II molecules.

## Competing interests

The authors declare that they have no competing interests.

## Authors' contributions

Method development: YX SZ. Conceived and designed the experiment: YX SZ. Performed the experiment: YX CL. Designed the web site YX. Analyzed the data: YX MQ XH SZ. Wrote the paper: YX XH SZ.
